# Access to high cost medicines in Australia: ethical perspectives

**DOI:** 10.1186/1743-8462-5-4

**Published:** 2008-05-19

**Authors:** Christine Y Lu, Paul Macneill, Ken Williams, Ric Day

**Affiliations:** 1 Department of Ambulatory Care and Prevention, Harvard Medical School, Boston, Massachusetts, USA; 2 Centre for Values, Ethics & the Law in Medicine, Faculty of Medicine, University of Sydney, Australia; 3 Faculty of Medicine, University of New South Wales, and Department of Clinical Pharmacology and Toxicology, St Vincent's Hospital, Sydney, Australia

## Abstract

Access to "high cost medicines" through Australia's Pharmaceutical Benefits Scheme (PBS) is tightly regulated. It is inherently difficult to apply any criteria-based system of control in a way that provides a fair balance between efficient use of limited resources for community needs and equitable individual access to care. We suggest, in relation to very high cost medicines, that the present arrangements be re-considered in order to overcome potential inequities. The biological agents for the treatment of rheumatoid arthritis are used as an example by which to discuss the ethical issues associated with the current scheme. Consideration of ethical aspects of the PBS and similar programs is important in order to achieve the fairest outcomes for individual patients, as well as for the community.

## Background

The goal of health care, stated in the Australia's National Medicines Policy, is to achieve optimal health and economic outcomes for society as a whole as well as for the *individual *patient [1]. Within the National Medicines Policy, the Pharmaceutical Benefits Scheme (PBS) aims to ensure affordable access to a wide range of prescription medicines equitably across all medical areas for Australians [2]. How health or pharmaceutical expenditure should be allocated is a very specific example of the general issue of distributive justice [3]. Healthcare payers world-wide cannot provide every medicine for all citizens without limitations, particularly for a national health system such as that found in Australia, where health services are heavily subsidised by tax revenues. "High cost medicines" (HCMs), predominantly products of the molecular biology revolution, have highlighted this dilemma. The concept of HCMs has not yet been clearly defined internationally. In Australia, HCMs have been described by Victorian public hospitals as medicines whose acquisition cost is greater than AUD$10,000 per patient per treatment course [4]. While HCMs have not been explicitly defined within the PBS it is important to note that if the total use of a drug is expected to cost more than AUD$10 million a year, its subsidy needs approval by the Commonwealth Department of Finance and Administration, or by the Cabinet [5]. This requirement would apply to, in relative terms (i) drugs of low unit cost but high utilization and (ii) drugs of high unit cost but low utilization. Most biological medicines are within the latter category.

Although the price of a medicine submitted for PBS subsidy ('listing') is an important issue, its cost-effectiveness is a more critical question to be answered in the PBS decision-making process. In an attempt to balance the benefits, risks and costs, PBS-subsidised access to medicines, including high-cost, biological medicines, is targeted to patients with the greatest capacity to benefit. The aim is to achieve an acceptable cost-effectiveness ratio (incremental cost per unit of incremental benefit). For medicines that are of high cost per patient, patients with the greatest capacity to benefit are likely to be a subset of patients with more advanced and active disease who have not been adequately controlled using less expensive, generally older and more established therapies. Provision of HCMs through the PBS is a significant achievement from the perspective of patient care and is concordant with the ethical principle of fairness. An effect, however, is that complex requirements for access to HCMs give the funding body an increased influence over clinical practice.

There has been little consideration of the ethical dimensions of such schemes by which we mean considering the fairness or equity of the policy and its application. In this paper, we examine, from an ethical perspective, recent approaches used in Australia to govern access to HCMs, using the example of the biological medicines ("biologicals") for the treatment of rheumatoid arthritis (namely, etanercept, infliximab, adalimumab, and anakinra). The anti-rheumatic biologicals are expensive (high cost per patient per year), they exhibit high cost-effectiveness ratios, their cumulative expenditure is likely to be high because rheumatoid arthritis is a relatively prevalent chronic condition, and there are uncertainties regarding their longer-term safety (such as risks of lymphoma and rare, serious infections). For these reasons, they provide a representative illustration of ethical issues associated with the current scheme.

## Discussion

### Prioritising access to high-cost medicines: the Australian approach

The Pharmaceutical Benefits Advisory Committee (PBAC) advises the federal Minister for Health about which medicines should be subsidised by the PBS. In making its recommendation, the PBAC considers the clinical and cost effectiveness of the medicine in relation to alternate therapies from a societal perspective [6]. That is, the PBS ensures reasonable value-for-money for Australian patients and taxpayers. Even though the PBS has an uncapped budget, overall government resources allocated to health care are limited for the possible range of diseases.

Requirements for access to HCMs (as here defined) under the PBS, in general, include the presence of 'severe active disease', 'molecular markers', and specified measures in follow-up assessment (Table 1) [7]. The criteria are based primarily on evidence from randomised controlled trials. In principle, this evidence-based approach to decision-making enables decision makers, clinicians and patients to make more informed decisions and to use resources more effectively [8-11]. However, benefits will not always extend to all individuals when access to medicines is rationalised according to 'average' responses observed in clinical trials. Additionally, patients who are participants in clinical trials are a small, and often not a closely representative, sample of the population of patients affected by the disease.

**Table 1:** Common requirements of access to high cost medicines under the PBS [15]

	**"Authority prescription" requirements**
**Criteria for initiating treatment**	• Severe active disease
	• Presence of "molecular markers" that predict a good treatment outcome
	• Failure to achieve adequate response to a step-up sequence of cheaper existing therapies
	• Patients required to sign a 'patient acknowledgement form'
**A patient agreement process**	• A Patient Acknowledgement Form to be signed by patients to acknowledge that treatment will only continue if the predetermined response criteria are achieved at follow-up assessment (e.g. 12 weeks for biological anti-rheumatic agents)
**Criteria for continuing treatment**	Clinical outcomes are evaluated according to predetermined quantifiable criteria at follow-up assessment
**Restricted prescribing rights**	• Prescription only by specialist physicians (e.g. rheumatologists initially for biologicals for rheumatoid arthritis. Prescribing rights were extended to clinical immunologists with expertise in the management of rheumatoid arthritis as of February 2004)
**Risk sharing arrangement **	• Price-volume agreement between sponsor and the government

Economic evaluation is increasingly used to prioritise provision of treatments for different medical conditions in response to burgeoning demands for health care. In general, clinicians tend to favour an *individual *rights-based view of ethics. By contrast, the goal of economic evaluation is *utilitarian *and relates to outcomes for the whole population [3]. Economic evaluations primarily focus on efficiency, thereby promoting *overall *welfare for the majority [3]. Whilst the majority of patients may benefit, not all individuals will. Economic analyses can override concerns for the individual patient such that goals of treatment, rational selection of treatments, and the individual patient's experience and perspective are ignored [12,13]. The limitations of making inferences from population-based evidence to individual patients, are not only relevant to the PBS approach, but are inherent in the "formulary" decisions of any organisation made on the basis of evidence from clinical trials and economic evaluations.

Likelihood of success (or capacity to benefit) is a necessary criterion from the perspective of distributive justice because a scarce resource should be distributed to patients who have a reasonable chance of benefit [3]. Targeting access to those with the greatest likelihood of success (for whom it represents good value for money) is used as a rational and equitable approach to deal with the difficult matter of access to medicines including HCMs under the PBS. This approach has increasingly relied upon 'molecular markers' or genetic information for individual patients. Examples of drugs whose funding by the Australian government already requires individual genetic information include: trastuzumab for the treatment of breast cancer (HER2 gene testing); imatinib for patients with chronic myeloid leukaemia (expression of the Philadelphia chromosome or the transcript, bcr-abl tyrosine kinase); and gefitinib for patients with non-small cell lung cancer (activating mutation of the epidermal growth factor receptor gene in tumour biopsy) [14,15].

Initially, the surrogate marker, rheumatoid-factor, was a criterion for access to the biologicals for treating rheumatoid arthritis [16], the PBAC having concluded that rheumatoid-factor was a 'treatment effect modifier' predictive of those most severely-affected and with a higher potential to respond [17]. Additionally, PBAC noted that this 'criterion' had been accepted by the representative rheumatologists from the Australian Rheumatology Association engaged in the stakeholder consultation process and by the sponsors [17]. However, this view was based largely on unpublished data held by the sponsor companies and was not supported by published literature [18,19]. Consequently, this requirement was subject to intense debate [17-19]. The rationale for this decision was unknown because all documents submitted to PBAC are considered 'commercial-in-confidence' by the pharmaceutical industry. The PBAC was not able, therefore, to defend its decision by tabling the data upon which its decision was based [17]. As a result, clinicians were unable to explain or defend this restriction to patients who were excluded from the treatment because of this criterion. The important responsibility of clinicians, who are, in effect, the 'gatekeepers', to manage the expectations of patients as well as their disease was thus impaired. Additional data were subsequently provided to the PBAC by the sponsor [20]. The criterion was removed as of June 2005 as an acknowledgement that the 'continuation rule' would cover the issue of 'inadequate' response [21]. The 'continuation rule' requires clear evidence of a substantial response to justify continued access to HCMs. The PBAC must be acknowledged for its commitment to address such problems and its willingness to consider new and additional data. However, the delay and difficulty in amending the PBS criteria highlights the issue that lack of transparency was fundamental to the concerns expressed by clinicians and patients about this decision. The transparency of PBAC decisions has improved markedly since then (as is discussed below).

Procedural justice is concerned with making and implementing decisions according to fair processes, that is, despite the absence of prior agreement on principles, a fair process yields a just outcome [22]. It requires transparency and inclusive decision-making processes [22]. These elements of procedural justice are fundamental to a sense of community ownership and can offset criticism of the 'economic analysis' based approach to determining access criteria.

In the context of priority-setting in Australia, the PBAC bears the final and legislative responsibility for recommending the access criteria for PBS medicines. Nevertheless all stakeholders should share in the process of establishing evidence-based criteria for access. Increasingly, the PBAC interacts with stakeholders to gain input to and acceptance of its decisions [6]. For example, a unique stakeholder collaboration between the PBAC, sponsor companies, and a small group of rheumatologists (the Australian Rheumatology Association Therapeutics Committee) contributed to the process for subsidising the anti-rheumatic biologicals [6,23]. It has been suggested that more structured and inclusive consultation, and increased participation by consumer representatives and patients in future processes would further enhance the fairness of the process and the system [24,25]. We believe it would also be valuable if PBS listing decisions and access criteria were subject to public input through a publicised, formal process of review to accommodate reasonable arguments or new evidence. Mechanisms for challenging and disputing decisions are necessary components to increase the fairness of the process [22].

Recent moves towards greater transparency of PBAC decisions are welcome developments [6]. An important milestone in this setting is the publication of 'Public Summary Documents' which have been available on the PBS website since late 2005. These documents now provide an outline of submissions (including a list of clinical trials of the medicine and results of key trials). Although details of the economic analyses remain unpublished, significant insights into PBAC decisions are now available through these documents. They are also useful for informing clinicians and patients about safe, effective and cost-effective use of the new drug in clinical practice.

Transparency also requires disclosure of potential conflicts of interest. This is critical for the effectiveness and acceptance of a trust-based stakeholder engagement process. Adequate access to unbiased, credible information and provision of training and support for stakeholders (particularly for clinicians, patients, carers, and the public) is likely to increase the ability of stakeholders to make informed contributions as well as to reduce any unbalanced influence of the industry on stakeholder perspectives. Furthermore, representatives engaged in the stakeholder collaboration process have the responsibility to effectively communicate the rationale behind decisions to their constituencies. Greater transparency, improved accountability, and increasing stakeholder involvement are all necessary in the effort to enhance public understanding of, support for, and community ownership of, PBS decisions. Again, these approaches to improving the decision-making process around access to medicines can be considered universal, applying to any organisation responsible for determining access criteria.

### Implementing the PBS restrictions

Three central goals of fairness are equity, efficiency, and accountability [26]. A basic requirement of justice is that those with equal needs have equal opportunities to access care. However, patients with equal needs do not have equal opportunities to access rheumatological services in Australia. Access to a rheumatologist varies considerably between the States and Territories and the PBS criteria require a rheumatologist to apply for the biologicals on behalf of the patient. Not surprisingly, analysis of prescription data by geographical location indicates that there is a correlation between utilisation of biological agents and the *per capita *ratio of rheumatologists [27,28]. Patients in remote or rural areas are, therefore, disadvantaged. A remedy is needed as the goal of fairness is not being achieved. Geographical impediments to legitimate access could be overcome in part, for example, by allowing general physicians in remote and rural areas to apply for access to HCMs for their patients.

Under the PBS, 'authority required' prescribing restrictions are usually employed as part of the access arrangements for HCMs. The 'authority required' mechanism is designed to limit the publicly funded use of medicines to those patients whose disease features and previous responses to therapy identify them as belonging to the subset of patients in whom the medicine has been shown to be cost-effective. Medicare Australia, the administering agency, is an important partner with the PBS in enabling patients' access to medicines. However, the access procedures to be followed are time-consuming for prescribers and patients, especially for HCMs where much information is requested by Medicare Australia. For example, there is difficulty locating records of laboratory tests and a detailed pharmacotherapy history for patients, many of whom have a long history of disease and have been under the care of multiple medical practitioners [25]. This problem is likely to diminish as patients with shorter histories of rheumatoid arthritis become eligible for these medicines. These requirements, however, are seen to be intrusive and a means of 'policing' inappropriate or over-use by prescribers [25]. The administrative burden is likely to reduce the amount of time physicians have to interact with their patients clinically. This issue has also been identified by pharmaceutical benefit management organisations in the United States [29]. Such procedures are also administratively cumbersome and costly for the PBS [25] thus reducing administrative efficiency.

Due to the significant cost of the biologicals and the uncertainties regarding their longer-term cost-effectiveness, it is reasonable to monitor patients closely and withdraw treatment promptly if it is not effective. This approach is also good clinical practice for any medicine, and the 'continuation rule' introduced by the PBS enforces this. Monitoring whether these indices of good prescribing practice are occurring at satisfactory rates along with evaluation of drug utilisation and health outcomes is a critical aspect of accountability for the PBS. Given the expenditure and the consequences of commencing or withholding critical treatments such as the biologicals, analysis of this data should be considered an ethical necessity and a wise use of public resources. Increased and facilitated access to de-identified, individual-level prescription and health outcome data but with appropriate protections of privacy in place is long overdue in Australia. Regular reporting of the outcomes achieved by programmes of access to HCMs is eminently sensible and should be considered a high priority [30].

Another innovation in PBS procedures around access to HCMs in recent years has been the requirement that an agreement be signed by patients to acknowledge that the drug treatment will continue only if there is a satisfactory outcome (or clinical response). However, in the case of the biologicals, a significant ethical concern has been the brevity of the written explanation in the Patient Acknowledgement Form. Clearly the patient has a right to be informed about the criteria that qualify him or her for initial and on-going access. Provision of explanatory material, or guidance to clinicians regarding how to address this issue with patients, would also be useful. Due to the complexity of the PBS-criteria, and the costs and potential risks of toxicity of biologicals, a brief information sheet is needed to improve understanding of the issues that are important to a patient seeking subsidised treatment.

### The way forward: Appeals on behalf of individual patients

Need and likelihood of success are both value-laden concepts. Individual patient's circumstances need to be considered in the context of the best available evidence. Patients near the margins of eligibility for access to a HCM but not quite meeting all the criteria (that is, evidence of cost-effectiveness in these sub-sets of patients may be insufficient or unclear) highlight the tensions that need to be examined. Consider a hypothetical scenario of a patient X with severe active rheumatoid arthritis despite optimal treatment with the available non-biological medicines. The new high-cost biological listed on the PBS is the next and only option but a criterion for access is not met – for example, an inflammatory marker – the erythrocyte sedimentation rate (ESR) – is lower than the level specified in the requirements for access (ESR > 25 mm/hour). The phenomenon of active disease with an unusually low ESR is well recognised, but such patients are not admitted to clinical trials because elevated ESR is often an inclusion criterion for such trials. In our example, patient X meets all other criteria, thus the drug is very likely to be as effective as for other patients who meet the criteria, with the 'continuation rule' governing follow-up clinical assessment and ongoing access. The argument for denying access is, therefore, not strong. At an ethical level, we argue that patients similarly near the criteria limits for eligibility for access to a HCM should be considered individually. This is because self-funding by most patients is not a realistic option (e.g., anti-rheumatic biological treatment costs about AUD$20,000 per patient per annum) and the uncertainty regarding factors that contribute to success (adequate clinical improvement) of treatment. For such patients, as in the example above, a formal, fair and transparent mechanism for appeal would go a long way to improve the public support for PBS. Similarly, treatment might be offered to a patient who has a diagnosis that overlaps considerably with the diagnosis for which access is allowed. The case to be made would be strong if there was good published evidence for efficacy in the overlapping condition and again the patient had exhausted all other evidence-based and reasonable options.

To some extent, pharmacists and medical advisors with clinical knowledge from Medicare Australia who assess applications for subsidised treatment have acted as a "review" panel. This is necessary because any system that relies on criteria, or the application of rules, needs some discretion in applying particular rules to the actual circumstances. In practice there is always some discretionary function in the application of criteria. We, therefore, accept that the system for making these judgments and the current functions within Medicare Australia is a good starting point. However, we suggest that it is preferable to establish a process that is open to public scrutiny. A streamlined appeals panel could be established with a few clinicians (say three) appointed by the Minister, who could meet (by telephone) to decide whether a particular patient should be eligible for subsidised treatment (such as the cases discussed above). The proposed system would be applicable to relatively few patients and unlikely to impose a large administrative burden and cost on the PBS. This process should be open, fair, consistent and accountable [22] rather than *ad hoc *and informal as is the current situation. Whilst we have suggested the outlines of a formal process, the details of its establishment and implementation require more thought and public discussion. Importantly however, the appeal mechanism must reflect the National Medicines Policy and the integrity of the PBS. Stakeholder involvement and ownership of the appeal process so developed will add to its acceptance.

While such a mechanism does introduce a further level of administration, its cost could be minimal. In any case the cost is justified by the need for a discretionary function and the potential for an open process to manage and reduce the tension between meeting the needs of the population and those of the individuals. We also believe that such a process could achieve an increase in equity. Even if the result of an appeal was the rejection of a request for access, the justification provided to prescribers, patients and representative groups would improve understanding. In itself, this would lead to increased acceptance of any decisions. Risk-sharing agreements (such as price-volume or rebate agreements) [31] between sponsor and government would limit financial exposure of the government and the taxpayer. These risk sharing instruments are a disincentive to any stakeholders who might be tempted to abuse the mechanism.

## Conclusion

Not everyone who has a rational case for subsidised access to a particular form of healthcare can gain access to it. This is because the assessment and establishment of 'cost effectiveness' of particular interventions relies on aggregated data collected as part of clinical trials, carried out in varyingly representative samples of the wider population affected by the disease. Some process is needed, therefore, to overcome these potential inequities and to enhance the fairness (and perception of fairness) of the system for accessing HCMs. Wider public debate to determine the fundamental principles and processes of targeted access to expensive medicines should, in our view, be a national priority. Provision of training and support is critical to achieve greater stakeholder involvement; physicians and patients are more likely to endorse criteria developed through an inclusive, transparent decision-making process. We advocate a formal mechanism for public input and review of PBS access criteria when additional, sound data become available. We also advocate exploring a defined mechanism to deal with the small number of individual patients who have exhausted all other treatment options despite PBS access criteria for a HCM not being met completely. These suggested embellishments to the PBS, we contend, are critical for ensuring procedural justice and will increase accountability and public confidence. The PBS in Australia is widely acknowledged to be outstanding [32,33]. The present proposals are offered to address issues we have identified involving and impacting on some individual patients who are excluded currently from subsidised access to HCMs when a rational case can be made to allow access. We believe that the adoption of these measures would enhance the equity of the Scheme in respect of patients with a clear and justifiable need and would ensure that the PBS continues to evolve and function ethically.

## Competing interests

CYL and PM have nothing to declare. KW has been a member of the Advisory Board to the sponsor for adalimumab. RD has been a member of the Advisory Board to sponsors for adalimumab, infliximab, and anakinra in Australia. KW and RD have also been contracted to undertake clinical trials of etanercept, infliximab, adalimumab, and anakinra. Recompense for these activities is placed in audited hospital trust funds for use in the research activities of the Clinical Pharmacology Department, St Vincent's Hospital, Sydney.

## Authors' contributions

CYL, KW, and RD have made substantial contributions to the conception and structure of the manuscript and drafting and revising the manuscript. PM has been involved drafting the manuscript and revising it critically for intellectual content. All authors read and approved the final manuscript.
